# Survival and transfer potential of *Salmonella enterica* serovar Typhimurium colonising polyethylene microplastics in contaminated agricultural soils

**DOI:** 10.1007/s11356-024-34491-4

**Published:** 2024-08-07

**Authors:** Luke Woodford, Rosie Fellows, Hannah L. White, Michael J. Ormsby, Chloe J. Pow, Richard S. Quilliam

**Affiliations:** Biological and Environmental Sciences, Faculty of Natural Sciences, University of Stirling, Stirling, FK9 4LA UK

**Keywords:** Flooding, Human pathogens, Leachate, Plastic pollution, Plastisphere, Soil quality

## Abstract

Agricultural environments are becoming increasingly contaminated with plastic pollution. Plastics in the environment can also provide a unique habitat for microbial biofilm, termed the ‘plastisphere’, which can also support the persistence of human pathogens such as *Salmonella*. Human enteric *Salmonella enterica* serovar Typhimurium can enter agricultural environments via flooding or from irrigation with contaminated water. Using soil mesocosms we quantified the ability of *S*. Typhimurium to persist on microplastic beads in two agriculturally relevant soils, under ambient and repeat flood scenarios. *S*. Typhimurium persisted in the plastisphere for 35 days in both podzol and loamy soils; while during multiple flood events was able to survive in the plastisphere for up to 21 days. *S.* Typhimurium could dissociate from the plastisphere during flooding events and migrate through soil in leachate, and importantly could colonise new plastic particles in the soil, suggesting that plastic pollution in agricultural soils can aid *S.* Typhimurium persistence and facilitate further dissemination within the environment. The potential for increased survival of enteric human pathogens in agricultural and food production environments due to plastic contamination poses a significant public health risk, particularly in potato or root vegetable systems where there is the potential for direct contact with crops.

**Supplementary Information:**

The online version contains supplementary material available at 10.1007/s11356-024-34491-4.

## Introduction

Annual global plastic production exceeds 300 million tonnes, and over 32% of all plastic waste ends up in the terrestrial environment (Kumar et al. 2020; Rillig and Lehmann 2020). The release of plastics into terrestrial environments is four to 23 times higher than in marine environments (Horton et al. 2017), yet the majority of research focuses on the impact of plastics in marine environments (Omeyer et al. 2022). Microplastics (1 µm–5 mm in size) originate from either a primary source, e.g. cosmetic products, or from secondary sources via fragmentation of larger plastics such as plastic mulching sheets used in agriculture (Zhou et al. 2023). Plastic polymers such as polyethylene (PE), polystyrene (PS), and polyvinylchloride (PVC) can enter soil systems from contaminated irrigation or floodwater, agricultural practices (e.g. mulching films and seed dressings), windblown from landfill sites, or contamination of water systems (Kumar et al. 2020; Ren et al. 2021; Steinmetz et al. 2016; Zhang et al. 2020). Livestock frequently ingest microplastics found in feed and feed bags, which subsequently enter agronomic systems via excrement, or via the application of organic fertilisers, manure, or composts (Beriot et al. 2021; Wu et al. 2021; Yang et al. 2021). Livestock faeces can also carry a range of zoonotic pathogens (including *Salmonella* spp. and *Escherichia coli*), which are capable of colonising microplastics (Ormsby et al. 2024a, b, c; 2023).

Once in the environment, plastics rapidly become colonised by microbial biofilms comprising complex microbial communities in a habitat known as the ‘plastisphere’ (Zettler et al. 2013). The plastisphere supports a diverse community of organisms shaped by the specific stressors of the external environment (Basili et al. 2020), and often differs from the surrounding microbial community (Martínez-Campos et al. 2021; McCormick et al. 2014). Soil directly surrounding plastics has been termed the ‘microplastisphere’ and provides a microbial habitat unique from both the rhizosphere and the surrounding bulk soil (Zhou et al. 2021). Differences in microbial community structure and diversity in bulk soil and around the plastisphere are influenced by the surface area, size, and type of plastic polymer, and the level of hydrophobicity (Zhu et al. 2022). Once in soil systems, microplastics can alter the composition of microbial communities (Li and Xiao 2023), leading to altered nutrient availability and carbon and nitrogen cycling. Importantly, the plastisphere commonly contains human pathogens, including viruses, bacteria, and fungi (Gkoutselis et al. 2021; Metcalf et al. 2022; Moresco et al. 2021), although the associated risks with human health are still debated (Beloe et al. 2022). The effects of microplastics in edaphic systems have thus far only focused on the impacts on physicochemical soil properties and changes to soil microbial communities (Ma et al. 2023; Zhang et al. 2020); however, little is known about the capacity of microplastics to act as vectors for human pathogens in soil (Quilliam et al. 2023; Zhu et al. 2022).

Human pathogenic *Salmonella* serovars, such as *Salmonella enterica* serovar Typhimurium, can cause systemic infections and accounts for ca. 594,000 cases and ca. 79,000 deaths a year (‘Global Burden of Disease Study’ 2019). *Salmonella* Typhimurium can enter soil systems via organic fertilisers and animal manure, flooding events, or via contaminated irrigation water; survival and persistence is subsequently influenced by soil type and fertiliser (Jechalke et al. 2019; Pornsukarom and Thakur 2016). It has recently been demonstrated that virulent strains of *S.* Typhimurium can persist on the surface of plastic bags in waste piles (Ormsby et al. 2024a), although how this relates to survival in other environments, particularly soil and agricultural systems, where there are clear pathways for human exposure, is unknown. Therefore, in this study, we aimed to (1) quantify the persistence of *S*. Typhimurium on low-density polyethylene (LDPE) microplastic beads in two contrasting agricultural soil types; (2) quantify the subsequent transfer potential of *S*. Typhimurium from colonised microplastics in simulated floodwater to uncolonised microplastics in soil; and (3) determine the potential for vertical transfer of *S.* Typhimurium from contaminated microplastics through the soil horizon as soil leachate.

## Materials and methods

### Soil and water

Two typical agricultural soil types (a podzol and a loamy soil) were selected based on their contrasting pH profiles. Both soils were dried at room temperature and sieved to 4 mm before being used. The pH and electrical conductivity (EC) of each soil were measured in distilled water (5:1 v:w) using a portable probe (Hanna Instruments Ltd., UK). Moisture content was calculated by oven drying at 105 °C and total organic matter was estimated from loss on ignition (LOI) using a Carbolite muffle furnace. Total nitrogen (N) and total carbon (C) were determined using a Flash smart NC-org -elemental analyser (Thermo Fisher, UK) (Table S1).

River water was collected from the Allan Water (Bridge of Allan, Scotland) in plastic carboys. Prior to use, river water and soils were screened for the presence of chloramphenicol resistant bacteria on Luria–Bertani (LB) agar (Invitrogen, UK) with chloramphenicol (25 mg/mL), with no growth detected after 24 h at 37 °C (data not shown).

### Biofilm generation and inoculation of plastic and glass beads

Low-density polyethylene (LDPE) plastic beads (Goodfellow, UK) and glass beads (Hecht Karl, Germany), both with a diameter of 4 mm, were placed in spherical stainless steel metal cages (45-mm high, 38-mm diameter, 1-mm pore size; Golf, China) and added to replicate glass tanks containing 7 L river water with additional trace metals (added as a 1 mL/L solution of CuCl_2_.2H_2_O, 15 mg/L; NiCl_2_.H_2_O, 25 145 mg/L; Na_2_MoO_4_.2H_2_O, 25 mg/L; ZnCl_2_, 70 mg/L; MnCl_2_.4H_2_O, 100 mg/L; CoCl_2_.6H_2_O, 120 146 mg/L; FeCl, 4 g/L; EDTA, 2 g/L; HCl [25%], 6.5 mL/L) (Eguchi et al. 1996) and aerated continuously using a pond air pump (Swell, UK). The tanks were covered with black plastic to prevent evaporation and left at ambient room temperature (ca. 18–21 °C) for 72 h to allow a natural biofilm to develop.

The pathogen used in all experiments in this study was an isolate of chloramphenicol-resistant *Salmonella enterica* serovar Typhimurium D23580 (hereby referred to as *S.* Typhimurium), which prior to use was grown in LB broth at 37 °C with shaking at 120 rpm, unless otherwise stated. An overnight culture of *S.* Typhimurium was centrifuged at 4000 rpm for 5 min, resuspended in 20 mL PBS, and added into each replicate glass tank (to give a final concentration of ca. 2.8 × 10^5^ CFU/mL). After 96 h, all the LDPE and glass beads were removed from the tanks and lightly rinsed in Milli-Q water (Millipore Milli-Q Synthesis System, USA) to remove lightly attached microbes not part of the plastisphere, and then weighed prior to being used in mesocosms.

### Design of soil mesocosms to quantify *S*. Typhimurium persistence

**Fig. 1:**
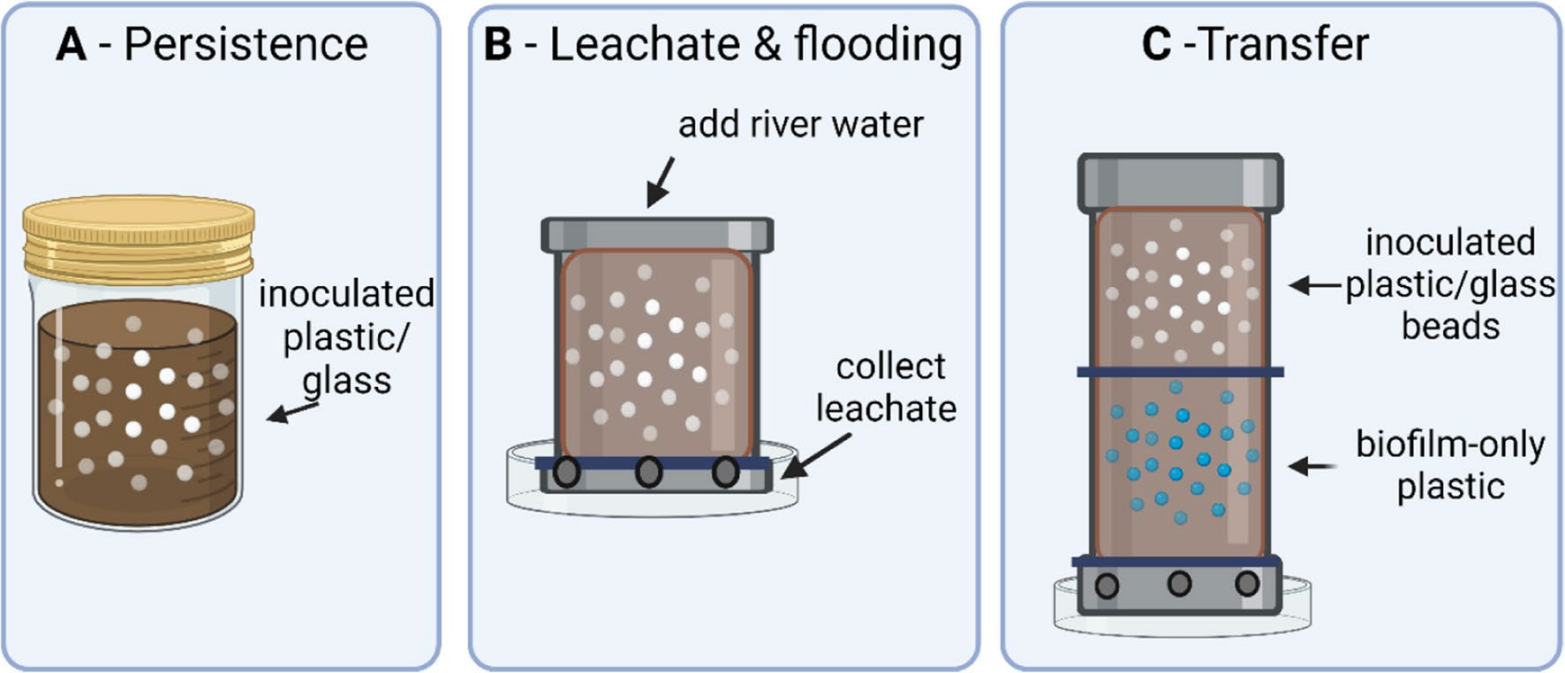
Schematic of the different experimental designs. **A** Persistence of *S*. Typhimurium on plastic and glass beads in different soil types; **B** flooding and soil leachate collection; **C** transfer of *S.* Typhimurium from inoculated plastic or glass beads to biofilm-only plastics through a soil column flooded with river water

Replicate sterile glass mesocosm jars (total volume 150 mL) contained 120 g of either podzol or loam soil and 3 g (2.5% w/w) of either plastic or glass beads inoculated with *S.* Typhimurium. Subsequently, 25 mL of river water was added to each mesocosm and the contaminated beads thoroughly mixed into the soil. An *S.* Typhimurium-only ‘culture control’ was made from an overnight culture diluted 1:100 in fresh, pre-warmed LB broth (to 37 °C), and grown to an approximate OD_600nm_ of 0.2. Twenty millilitres of this diluted culture was added to 2 L of river water to give a final concentration of ca. 10^6^ CFU/mL. From this, 25 mL was added to each ‘culture-control’ mesocosm containing 120 g soil, thoroughly mixed, and subsequently used as *S.* Typhimurium-only controls (Fig. 1A). All mesocosms were stored in the dark at room temperature (18–21 °C), with their lids loosely attached.

At each sampling time point (1, 2, 3, 4, 7, 10, 14, 21, 28, and 35 days), sterile forceps were used to remove all of the plastic and glass beads from the soil (four replicate mesocosms at each time point), and placed into individual sterile 30-mL glass universal tubes (one for each replicate mesocosm), mixed with 10 mL of Milli-Q water, inverted five times (to remove loosely adhering bacteria), and the liquid discarded; a fresh 5 mL of Milli-Q water was then added to each tube. For the ‘culture control’, 3 g of the soil was removed from each mesocosm and mixed with 5 mL Milli-Q water. Universal tubes containing LDPE, glass, or culture control samples were vortexed for 5 min at 1500 rpm to disrupt the biofilm. The Milli-Q water was then serially diluted in PBS and plated out on LB chloramphenicol media, incubated overnight at 37 °C and *S.* Typhimurium colony forming units (CFU) enumerated.

### Soil flooding and the subsequent transfer of *S*. Typhimurium from colonised plastic and glass beads

To understand the potential for plastic and glass particles to facilitate the survival and transfer of *S.* Typhimurium through flooded soils, two different mesocosm experiments were designed. Firstly, the effect of floodwater moving down through a soil column containing *S.* Typhimurium-contaminated plastic or glass particles was quantified in replicate mesocosms (plastic pipes, 10 cm × 6.5 cm, with 3 × 3 mm holes in the base, sterilised with 70% EtOH before use; Fig. 1B) containing 200 g of soil and 5 g (2.5% w/w) of LDPE or glass beads (colonised with a natural biofilm and inoculated with *S*. Typhimurium as described above) and mixed with 25 mL of river water. Replicate ‘culture-only control’ (no plastic or glass beads) mesocosms consisted of an overnight culture of *S.* Typhimurium added to 25 mL river water and mixed with the soil. Stainless steel wire mesh (0.125-mm mesh size; Amazon, UK) was placed around the base of each mesocosm to retain the soil but allow the leachate to flow through (Fig. 1B). At each designated time point (1, 2, 3, 4, 7, 10, 14, and 21 days), 50 mL of river water was added to the top of each pipe and the leachate collected in a Petri dish. Mesocosms were typically left for approx. 4 h to allow sufficient time for leachate to run through. Viable cells of *S*. Typhimurium in the leachate were subsequently enumerated on LB chloramphenicol media as described above.

Secondly, we quantified whether *S*. Typhimurium could be transferred from *S*. Typhimurium-colonised plastic and glass beads through soil and colonise new plastic beads. To achieve this, mesocosms were designed whereby the top compartment contained plastic or glass beads (10 g equal to 5% w/w) colonised by a natural biofilm and contaminated by *S.* Typhimurium (10^4^–10^5^ CFU/g) or a ‘culture-only control’ (culture mixed with 200 g of soil). The bottom compartment of each mesocosm contained 200 g of soil mixed with 5 g (equal to 2.5% w/w) of LDPE plastic beads colonised by a natural biofilm (generated as described above). The two halves of each column were partitioned using a stainless-steel mesh (0.125-mm pores) to retain the soil but allow the transfer of liquid; all mesocosms were positioned in Petri dishes to collect leachate (Fig. 1C). At each time point (1, 2, 3, 4, 7, 10,and 14 days), 50 mL of river water was poured through four replicate columns for each treatment in each soil type (24 total) and the leachate was discarded. At 7 and 14 days, the columns were disassembled and the LDPE and glass beads in the top compartment, and the plastic beads in the bottom compartments were carefully removed with sterile forceps. For the ‘culture-only control’, 5 g of soil was removed from the top of each replicate mesocosm. The concentration of *S*. Typhimurium was determined as described above. The same weight of soil (5 g) was removed from the bottom compartment and analysed by the same method (data not shown).

### PCR confirmation of *S*. Typhimurium

PCR of the *ttr* gene (encoding tetrathionate reductase; *ttr_*for: 5′-CTCACCAGGAGATTACAACATGG-3′; ttr_rev: 5′-AGCTCAGACCAAAAGTGACCATC-3′ (Hopkins et al. 2009)) was conducted on a selection of colonies throughout the study, with all samples testing positive for *Salmonella*. Briefly, reactions consisted of a 2 × master mix (Qiagen, Germany), 0.4 µM forward and reverse primer, and a single colony, made to a final volume of 25 µL using nuclease-free water. Cyclic conditions of 95 °C for 1 min, 60 °C for 1 min, and 72 °C for 1 min were run for 30 cycles, with an initial 5 min at 95 °C and final extension of 72 °C for 5 min. All samples were analysed by 1% gel electrophoresis.

### Statistical analyses

Differences in *S.* Typhimurium concentrations between time points in the simulated soil flooding mesocosm experiment were assessed by one-way analysis of variance (ANOVA) and a Tukey post-hoc multiple comparison test (GraphPad Prism Software v. 9.5.1). To calculate *S.* Typhimurium die-off rates, CFU concentrations were normalised by transforming to log_10_ CFU/mL or log_10_ CFU/g. Linear regression analysis, carried out in Minitab version 18 (Minitab Inc.; State College, PA, USA), was used to describe the dynamics of *S.* Typhimurium die-off as previously described (Ormsby et al. 2024c, 2024a). Briefly, a log linear regression model was fitted to the log_10_ transformed data, which is described by the equation

$$\text{Log}10\left(C\right)=\text{Log}10\left({C}_{0}\right)-kt$$

where *C*_0_ is the cell concentration at *t* = 0 and *k* is a die-off rate constant (day minus 1).

The percentage decrease in *S*. Typhimurium concentration per unit time was determined as constant using the log linear model. Following a log-linear die-off profile, decimal reduction times (*D*-values; the number of days to reduce viable bacteria by 90%) were calculated based on the decline rates for each population. Analysis of variance (ANOVA) was used to assess the effect of different material types on *K* values, and Tukey post-hoc tests were used for comparing means. Pearson correlation coefficients were used to investigate the linear correlation between the *K* value and *D* value from the linear decline rate analysis for each material. From this, 95% confidence ellipses were calculated and generated using R (Kassambara 2020; R Core Team 2021).

## Results

### Persistence of *S*. Typhimurium in the plastisphere of two different soil types

**Fig. 2:**
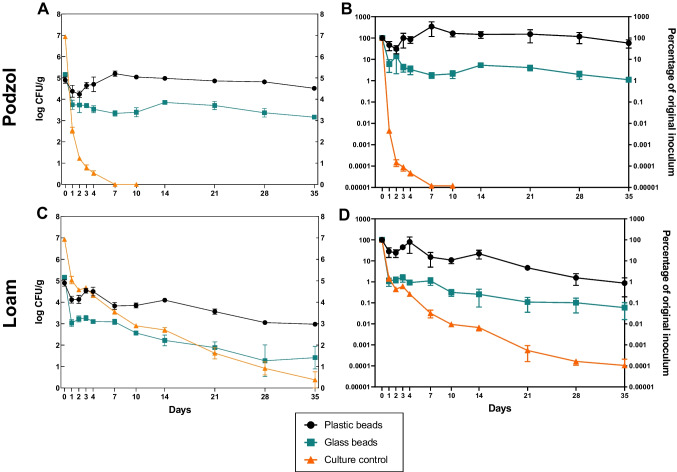
Persistence of *S.* Typhimurium on plastic and glass beads in different soil types. *S.* Typhimurium was enumerated from the surfaces of plastic beads and glass beads, and from a ‘culture-only control’ recovered from podzol (**A**, **B**) and loam (**C**, **D**) soils. Persistence is presented as the log CFU/g (**A**, **C**) and the percentage survival compared to the starting inoculum (**B**, **D**). Data points represent the mean (*n* = 4) ± SEM

*S.* Typhimurium was able to persist on the surface of both plastic and glass beads in podzol and loam soil. After 35 days, the concentration of *S.* Typhimurium persisting on the plastic beads was higher in both soil types compared to the glass, with 5 × 10^4^ CFU/g (58% of the original inoculum) and 1 × 10^3^ CFU/g (0.9% of the original inoculum) on plastic beads in the podzol and loam, and 2 × 10^3^ CFU/g (1.1% of the original inoculum) and 1 × 10^2^ CFU/g (0.06% of the original inoculum) on glass beads in the podzol and loam (Fig. 2). In the mesocosms containing ‘culture-only control’, no colonies were recovered in the podzol soil from 7 days onward, although *S*. Typhimurium was still detectable at 4 × 10^1^ CFU/g (0.0001% of the original inoculum) after 35 days in the loam soil.

Log linear regression models were used to determine the linear decline rate constants (*K*) and decimal reduction times (*D*-values) of *S.* Typhimurium colonising plastic and glass beads, or in the culture-only control. Both the soil type and the type of colonised material influenced the rate of decline, with *S.* Typhimurium *D*-values of 49.5 and 22.3 for podzol and loam, respectively, on the plastic beads, compared to *D*-values of 0.7 and 6.1 for the ‘culture-only control’ in podzol and loam, respectively (Table S2). The *K* value was significantly higher (*P* < 0.0001) for *S.* Typhimurium in both soil types for the ‘culture-only control’ compared to the *S.* Typhimurium concentration on plastic or glass, indicating a more rapid rate of decline when not associated with a biofilm on plastic or glass beads (Fig. S1).

### Persistence of *S*. Typhimurium on plastic and glass beads after flooding

**Fig. 3:**
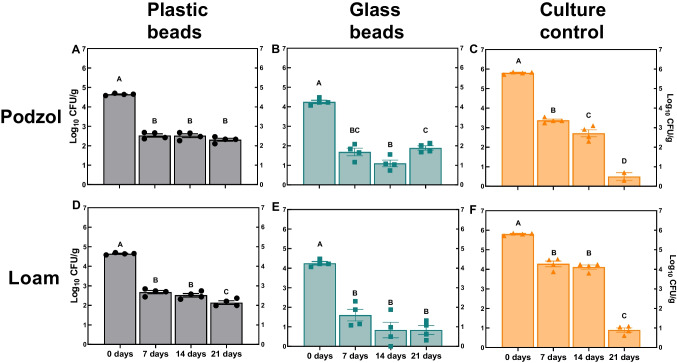
The influence of plastic and glass beads on the persistence of *S*. Typhimurium exposed to repeated flooding events. *S.* Typhimurium was enumerated after multiple flood events in podzol (**A**–**C**) and a loamy soil (**D**–**F**). Each bar represents the mean of four replicates ± SEM. Bars with different letters are significantly different to each other (*P* < 0.01)

Persistence of *S*. Typhimurium bound to plastic and glass beads or in ‘culture controls’ mixed into the soils was quantified after repeatedly flooding each mesocosm with river water. There was no difference in the concentration of *S.* Typhimurium recovered from the surface of the plastic beads in the podzol between days 7 and 21; although there was a significant decrease in *S.* Typhimurium on the plastic beads between days 14 and 21 in the loam soil (*P* < 0.01) (Fig. 3A and D). In the ‘culture only’ control, the concentration of *S.* Typhimurium decreased significantly at each time point in the podzol (*P* < 0.001) and decreased between 14 and 21 days in the loam soil (*P* < 0.001) (Fig. 3C and F). On the plastic beads there was a lower linear decline *K* value and higher *D* value for *S.* Typhimurium in both soil types compared to the glass beads or the ‘culture control’ (Table S3). There was a significant difference in *K* values (*P* < 0.0001) for *S.* Typhimurium concentration in the ‘culture control’ and the concentration of *S*. Typhimurium on inoculated glass and LDPE beads, with a strong negative Pearson correlation coefficient between *K* and *D* values for all samples (Fig. S2).

### Transfer of *S*. Typhimurium from plastic beads in floodwater to uncontaminated plastic beads in soil

**Fig. 4:**
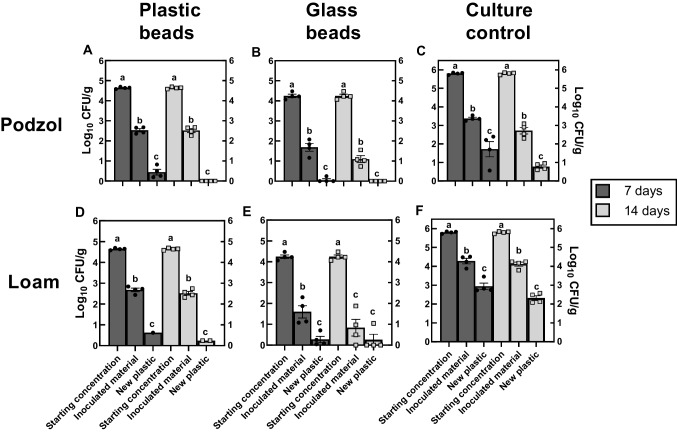
Transfer of *S.* Typhimurium to new plastic beads following a simulated flooding event. The ‘starting concentration’ represents colonisation of *S*. Typhimurium on plastic, glass, and in the ‘culture control’ inoculated soil prior to the experiment beginning. The ‘inoculated material’ is the concentration of *S.* Typhimurium on the plastic and glass beads or in the soil for the ‘culture only’ control after the specified period. ‘New plastic’ refers to the *S.* Typhimurium concentration on the new plastic beads placed in the bottom half of the mesocosm. Each bar represents the mean of four replicates ± SEM. Different letters above the bars indicate significant differences (*P* < 0.01) from ANOVA and Tukey post-hoc test

*S.* Typhimurium colonising plastic or glass beads in both soil types were able to detach from the biofilm and be transported through the soil following a simulated flooding event and subsequently colonise new plastic beads in the bottom compartment of the column after 7 days in the flooded soil (Fig. 4A, B, D, and E). However, after 14 days in the flooded soil, *S.* Typhimurium was only detected on plastics in the bottom compartment of the mesocosms containing loam soil (Fig. 4D). In the mesocosms containing the ‘culture control’, *S.* Typhimurium was also transferred through both soil types during simulated flooding and readily colonised the new plastics in the bottom compartment in the podzol and loam soils (Fig. 4C and F).

### Concentration of *S*. Typhimurium in soil leachate following simulated flooding events

**Fig. 5:**
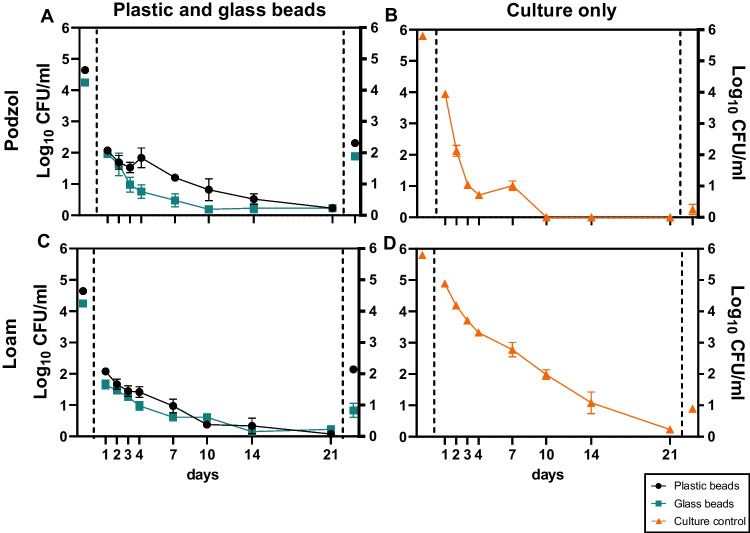
Recovery of *S.* Typhimurium in soil leachate. The concentration of recovered *S.* Typhimurium from plastic and glass in podzol (**A**) and loam (**C**) and the culture control in podzol (**B**) and loam (**D**). The data points on the far left of the dashed line are the concentrations of *S.* Typhimurium colonising the plastic and glass beads at day zero (**A**, **C**), and the data points on the far right of the dashed line are the concentrations of *S.* Typhimurium remaining on the plastic and glass beads after 21 days (**A**, **C**); or the concentration of the inoculum added to the soil at day 0 and the concentration remaining in the soil at the end (**B**, **D**). Each data point represents the mean of four replicates ± SEM

Within 24 h, *S.* Typhimurium was detectable in leachate from both soil types in all mesocosms (Fig. 5) and remained detectable throughout the 21 days of the experiment, albeit at low concentrations (e.g., by day 21, less than 1 Log_10_ CFU/mL recovered from any of the replicates). In the ‘culture-only’ control in the loam soil, *S.* Typhimurium also continued to be detected in the leachate for up to 21 days; however, by day 10 in the podzol leachate *S.* Typhimurium was no longer detected. Despite similar concentrations of *S.* Typhimurium being recovered in the leachate from mesocosms containing colonised plastic or glass beads, the final concentration of *S.* Typhimurium was higher on the plastic beads compared to the glass beads in both soil types (Fig. 5). However, the rate of decline (*D*-value) for the *S.* Typhimurium in the leachate was slowest for those cells that dissociated from the glass and plastic beads in the podzol (15.8 and 11.2 days, respectively), while in the loam soil the *D*-value for *S.* Typhimurium that dissociated from the glass beads was the same as the ‘culture control’ (4.2 days), while for the plastic beads it was higher (7.9 days) (Table S4; Fig. S3).

## Discussion

### The plastisphere can provide a protective environment for *S*. Typhimurium in soil

This study has demonstrated that the plastisphere provides significant protection for *S.* Typhimurium in the soil environment, allowing it to persist in two contrasting soil types at significantly higher concentrations than when added directly to soil. Furthermore, *S.* Typhimurium cells were subsequently able to dissociate from the plastisphere and transfer to surrounding soil, which demonstrates the potential co-pollutant risk of plastics and human pathogens in soil systems. *S.* Typhimurium was detectable in loamy soil for at least 35 days, although when added directly to the podzol, *S*. Typhimurium rapidly became undetectable, which was likely a response to the acidic pH of this soil type. The ability of *Salmonella* spp. to form biofilms and survive in the soil environment is influenced by soil moisture, pH, and mineral content (Alegbeleye et al. 2018), but being in the plastisphere can increase the persistence of *S.* Typhimurium and allow it to survive at significantly higher concentrations in both soil types.

The dynamic and cyclical (re)contamination of plastics in agricultural soils from either contaminated floodwater or irrigation water has important implications for environmental and human health risks. The pathogenic potential of soils used for crops or livestock could be significantly increased if they are contaminated with plastic pollution (Quilliam et al. 2023), particularly if this is acting as a protective reservoir for *S*. Typhimurium. As crops are being increasingly grown in microplastic contaminated soils (Chen et al. 2020; Li et al. 2022), potential human health risks could be further compounded by the facilitated increased survival of human enteric pathogens in agricultural environments with potential consequences for crops, livestock, and food safety.

### Persistence of *S*. Typhimurium in the plastisphere during flooding and further dissemination into the environment

The plastisphere aided the persistence of *S.* Typhimurium when the soil was repeatedly flooded, compared to *S.* Typhimurium inoculated freely in the soil, likely due to the protection biofilms provide bacterial communities from environmental stressors (Yin et al. 2019; Zhu et al. 2022). Flooding of soils can affect the availability and composition of nutrients, which together with changes in soil physical properties can have a significant impact on *Salmonella* spp. survival (Jechalke et al. 2019; Peng et al. 2022). In the podzol, the freely inoculated *S.* Typhimurium reduced in concentration quickly compared to when bound to the plastisphere, due to being washed from the soil by the repeated addition of flood water. Soils with high sand content typically have lower cation exchange capacity (CEC) compared to clay-based soils, as clay soil particles are negatively charged and hold on to cations in the soil. Podzols are less likely to retain floodwater compared to loamy soils because of the difference in CEC, which may reflect the differences in *S.* Typhimurium persistence when freely inoculated into these two soils.

The two different soil types will also contain contrasting concentrations of microbial biomass that may respond differently to waterlogging (Khan et al. 2022). During severe flooding, soil enzyme activity often increases, probably due to the addition of organic matter and detritus in floodwater (Macé et al. 2016). The lower CEC of podzols, e.g., due to a higher proportion of sand, make them less likely to retain water and more likely to have a reduced microbial biomass, resulting in potentially harsher conditions for the survival of human pathogens. Microbial biomass, the availability of nutrients, and extracellular enzyme activity can also be affected by microplastics in the soil (Zhang et al. 2024); therefore, a combination of flooding and plastic contamination could lead to a hostile environment for the survival of enteric pathogens not associated with the plastisphere.

Soil leachate collected from the contaminated plastics in both soil types still contained viable *S.* Typhimurium, which indicates that *S*. Typhimurium can dissociate from the plastisphere and be washed through the soil profile. This demonstrates that microplastics can not only simultaneously increase the duration of *S*. Typhimurium persistence in soil but also facilitate their further dissemination within the environment. Soil type can influence the movement of human pathogens, with loamy soils less likely to facilitate the rapid movement of floodwater compared to a podzol, which will have a higher volume of small pores and a larger total pore area (Lipiec et al. 2018). As well as affecting the movement of water, soil porosity and texture will also influence the ability of the microbial community to access nutrients through the pore network (Patel et al. 2021). Following the dissociation of *S.* Typhimurium from the plastisphere during flooding and subsequent waterlogging of soil, it could be rapidly transported through the soil and into waterbodies, further increasing its contamination potential (Callahan et al. 2017).

In this study, LDPE plastic beads were added to soil at 5% w/w, which is reflective of the quantity of plastic found in some agricultural settings (Palansooriya et al. 2022). As they are typically lightweight, microplastics can decrease soil bulk density, influence soil texture, and increase soil aeration (De Souza MacHado et al. 2018), all of which will contribute to the rapid movement of water through soil during flooding. This can result in mineral depletion of soil, with lower concentrations of total organic C and N in flooded soils (Saint-Laurent et al. 2014); however, whether repeated flooding and soil saturation reduced the survival of *S.* Typhimurium inoculated directly into the soil remains unclear. Compositional differences between the two soil types can also influence the movement of *S.* Typhimurium during flooding. When applied to different soil types as part of a liquid manure, *S.* Typhimurium was more likely to be found in the top 20 cm of soil compared to lower depths in soils (Bech et al. 2010). The concentration of human pathogens in the top of the soil profile increases the risk of contact with the aerial parts of crops, e.g., due to splash from rain or irrigation water, but also the (re)colonisation of plastic waste at the soil surface.

### *S*. Typhimurium can be transferred from the plastisphere to uncontaminated plastics in a shared soil environment

When placed in soil directly contaminated with *S*. Typhimurium or with plastic beads contaminated with *S*. Typhimurium, uncontaminated plastics became colonised with the pathogen. As floodwater passed through the soil, it transported cells of *S.* Typhimurium through the soil profile and delivered them to the new plastic beads where they subsequently became incorporated into the plastisphere. This suggests that microplastics in agricultural soil, e.g., from degrading plastic mulches, can act as sinks for colonisation by human enteric pathogens that have become resuspended and disseminated by either flood or irrigation water. Such transfer to uncolonised microplastics in the field may lead to the enhanced persistence of human pathogens and an increased risk of contact with livestock or humans (Ormsby et al. 2024b).

Once disassociated from the plastisphere and transferred into the soil, *S.* Typhimurium survival will be influenced by autochthonous microbial communities, which will be different in the two soil types used in this study (Liao et al. 2021). Interactions occurring at the soil-plastic interface may affect the subsequent persistence of *S.* Typhimurium in the plastisphere. The micro-plastisphere can produce enriched microbial hotspots in soil due to the presence of bioavailable carbon compounds in plastics (particularly bioplastics) (Zhou et al. 2021) that could be used by *S.* Typhimurium, which can alter its carbon metabolism in response to locally available sources of carbon in the soil (Han et al. 2023). The colonisation of new plastics by *S.* Typhimurium following flooding may be influenced by the available C or specific microbial hotspots associated with these plastics, particularly if farmers are increasingly using bioplastics to cover and protect crops. The soil plastisphere can also be a hotspot for horizontal gene transfer (HGT) of antibiotic resistance genes (ARGs) (Rillig et al. 2019; Zhu et al. 2022). HGT within the plastisphere is particularly likely to occur when manure is added to the soil, or at elevated temperature or moisture (Zhu et al. 2022).

## Conclusions

The ability of *S.* Typhimurium to persist on plastics in the soil has significant implications for food production. Nano- and microplastics can bind to leaves and fruits of crops and be taken up by roots (Azeem et al. 2021), and the subsequent persistence of human pathogens in the plastisphere will determine the public health risk from both soils and crops (Quilliam et al. 2023), including the ability of pathogens to transfer from plastics onto the surface of edible salad leaves (Woodford et al. 2024). Microplastics represent a novel and rapidly increasing environmental concern in soil systems, particularly for agriculture. Plastics can alter soil physicochemical properties and autochthonous microbial communities and have the potential to deliver harmful human pathogens into the food chain. Given the lightweight and durable nature of plastics, pathogens found in the plastisphere could spread further and last longer than other potentially contaminated materials, posing a greater threat to human health.

## Supplementary Information

Below is the link to the electronic supplementary material.

Supplementary file1 (DOCX 450 KB)

## Data Availability

All data is either presented in the manuscript and supplementary files, or will be made available upon request.
