# Supplementary material for: Survival and transfer potential of Salmonella enterica serovar Typhimurium colonising polyethylene microplastics in contaminated agricultural soils
Source: Environ Sci Pollut Res Int. 2024 Aug 7;31(39):51353–63. doi: 10.1007/s11356-024-34491-4 (PMC11374834; doi:10.1007/s11356-024-34491-4)
Supplement: Supplementary file 1 — Supplementary file1 (DOCX 450 KB) [file 11356_2024_34491_MOESM1_ESM.docx]

**Woodford et al 2024 - Supplementary Information**

**Table S1: Properties of podzol and loam soils.** All values are the mean of four measurements taken from a composite soil sample.

| **Soil** | **Total N (%)** | **Total C (%)** | **pH** | **EC (µS cm^−1^)** | **Moisture (%)** | **LOI (%)** |
| --- | --- | --- | --- | --- | --- | --- |
| Loam | 0.24 | 4.453 | 6.7 | 17 | 21.20 | 9.03 |
| Podzol | 0.023 | 0.733 | 4.5 | 132 | 14.87 | 1.3 |

**Table S2:** **Linear decline rates and decimal reduction times for S. Typhimurium in podzol and loam soils**. Linear decline rate constant = 2.303 x slope gradient (calculated from Figure 3).

| **Sample** | | ***K* (day^-1^)** | | **D-value (days)** | **R^2^** |
| --- | --- | --- | --- | --- | --- |
|  |  | **Mean** | **SEM** |  |  |
| **LDPE** | Podzol | 0.048 | 0.005 | 49.5* | 0.732 |
|  | Loam | 0.104 | 0.004 | 22.3 | 0.607 |
| **Culture control** | Podzol | 3.350 | 0.119 | 0.7 | 0.766 |
|  | Loam | 0.388 | 0.035 | 6.1 | 0.813 |
| **Glass** | Podzol | 0.052 | 0.011 | 50.3 | 0.214 |
|  | Loam | 0.141 | 0.015 | 16.9 | 0.388 |

*D-value determined from day 7 for LDPE in the podzol due to the initial increase in concentration after 96 hours.

*
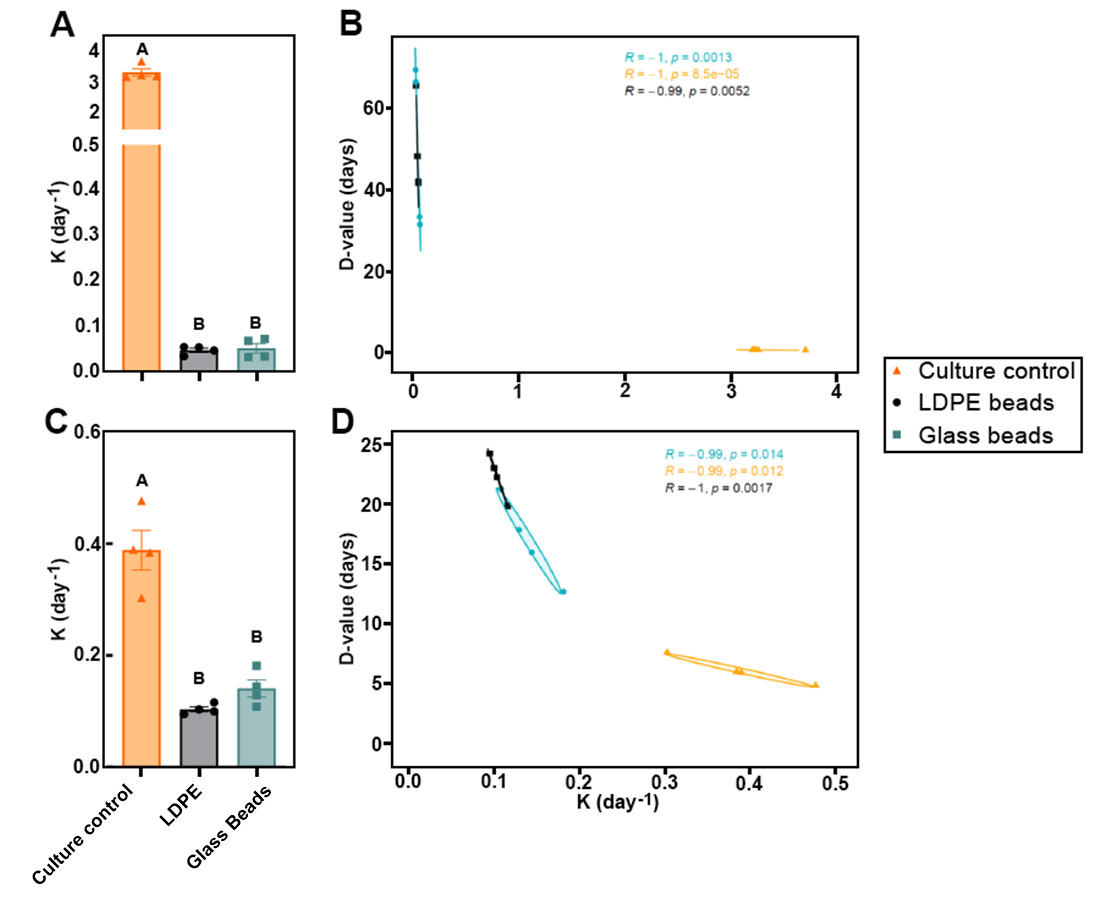
*

**Figure S1: Linear decline rates and decimal reduction times during *S.* Typhimurium persistence analysis.** The K value derived from the linear decline analysis (Table S3) is shown for LDPE beads, glass beads, and the ‘culture control’ in podzol (A) and loam (C). Analysis of variance (ANOVA) was used to assess the effect of material type on K values followed by a Tukey post-hoc multiple comparisons; different letters above bars indicate significant differences. The K value and D-value, derived from the linear decline analysis, were plotted against each other in podzol (B) and loam (D) with a Pearson correlation coefficient used to determine the correlation between the values (ellipses show 95% confidence).

**Table S3: Linear decline rates and decimal reduction times for *S.* Typhimurium in podzol and loam following multiple flood events**. Linear decline rate constant = 2.303 x Figure 4 slope gradient (not shown).

| **Sample** | | ***K* (day^-1^)** | | **D-value (days)** | **R^2^** |
| --- | --- | --- | --- | --- | --- |
|  |  | **Mean** | **SEM** |  |  |
| **LDPE** | Podzol | 0.231 | 0.008 | 10.0 | 0.669 |
|  | Loam | 0.252 | 0.013 | 9.2 | 0.768 |
| **Culture control** | Podzol | 0.526 | 0.022 | 4.4 | 0.919 |
|  | Loam | 0.490 | 0.012 | 4.7 | 0.863 |
| **Glass** | Podzol | 0.252 | 0.011 | 9.2 | 0.516 |
|  | Loam | 0.362 | 0.033 | 6.5 | 0.490 |

**
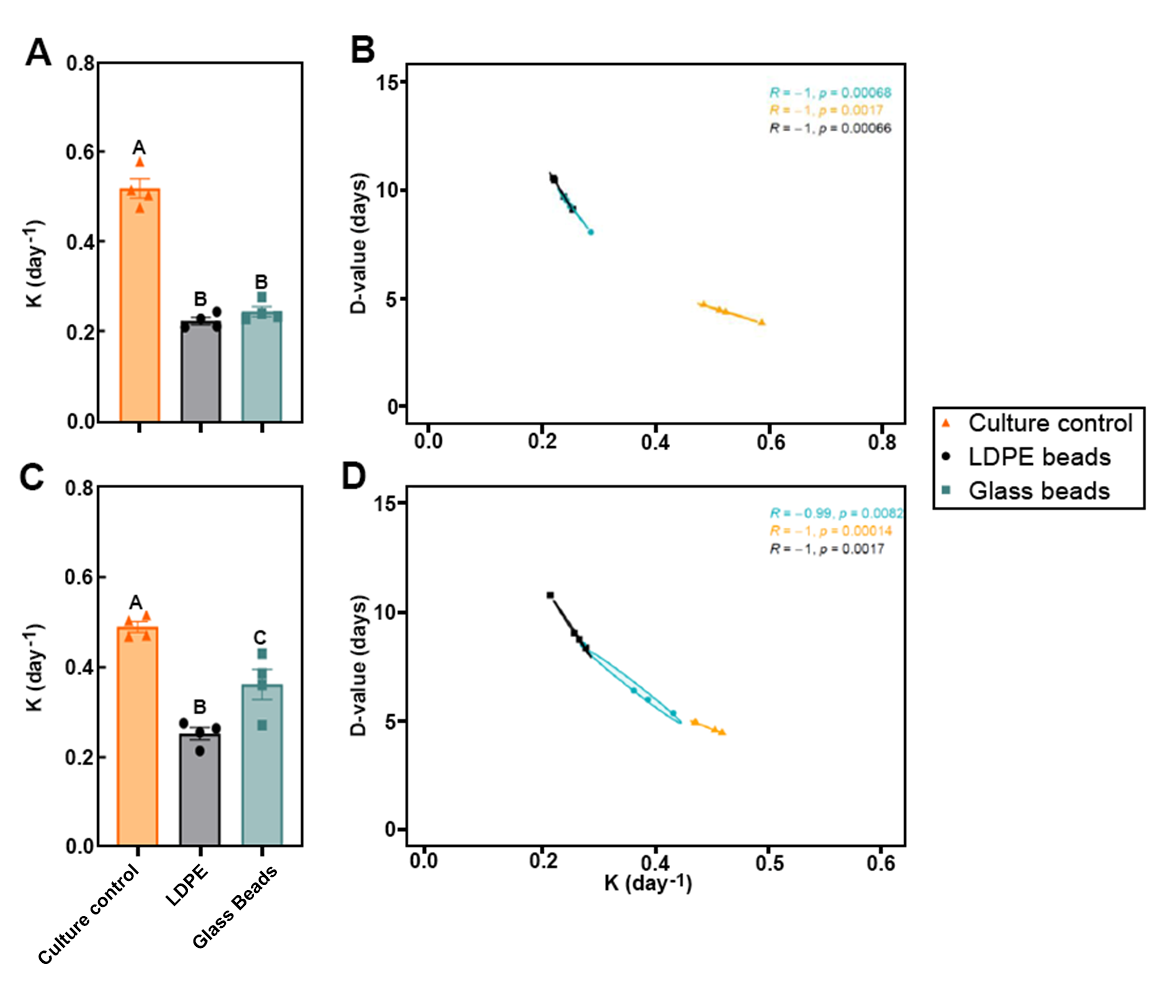
**

**Figure S2: Linear decline rates and decimal reduction times for *S.* Typhimurium during flooding analysis.** The K value derived from the linear decline analysis (Table S4) for LDPE, glass and culture control in podzol (A) and loam (C) is shown. Each bar represents the mean of four replicates ±SEM. Analysis of variance (ANOVA) was used to assess the effect of material type on K values followed by a Tukey post-hoc multiple comparisons; different letters above bars indicate significant differences. The K value and D-value, derived from the linear decline analysis, were plotted against each other in podzol (B) and loam (D) with a Pearson correlation coefficient used to determine the correlation between the values (ellipses show 95% confidence).

**Table S4: Linear decline rates and decimal reduction times for *S*. Typhimurium recovered from soil leachate**. Linear decline rate constant = 2.303 x Figure 5 slope gradients*.*

| **Sample** | | **K (day^-1^)** | | **D-value (days)** | **R^2^** |
| --- | --- | --- | --- | --- | --- |
|  |  | **Mean** | **SEM** |  |  |
| **LDPE** | Podzol | 0.220 | 0.036 | 11.2 | 0.755 |
|  | Loam | 0.307 | 0.040 | 7.9 | 0.804 |
| **Culture control** | Podzol | 0.955 | 0.039 | 2.4 | 0.505 |
|  | Loam | 0.561 | 0.065 | 4.2 | 0.952 |
| **Glass** | Podzol | 0.157 | 0.022 | 15.8 | 0.433 |
|  | Loam | 0.193 | 0.042 | 13.3 | 0.561 |


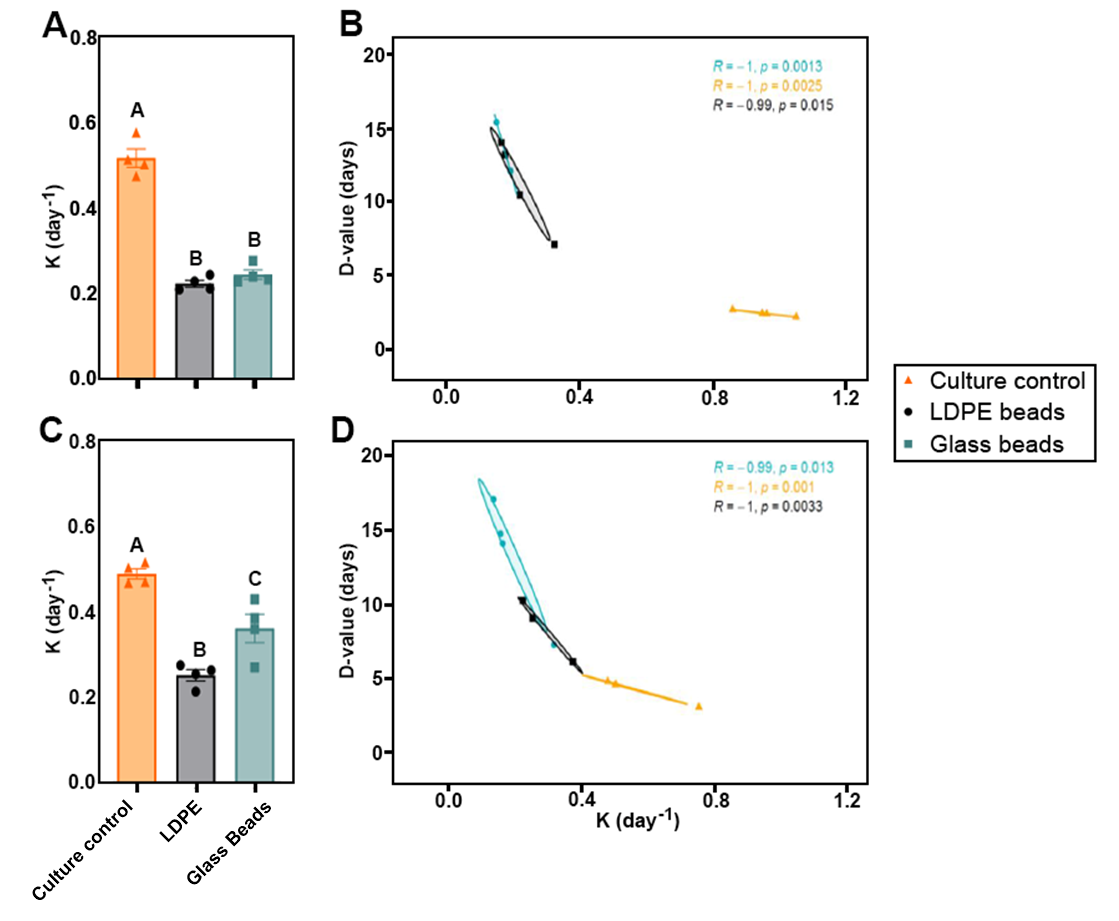


**Figure S3: Linear decline rates and decimal reduction times for *S.* Typhimurium recovered from soil leachate over time.** The K value derived from the linear decline analysis (Table S4) for LDPE, glass and ‘culture control’ samples in podzol (A) and loam (C) is shown. Analysis of variance (ANOVA) was used to assess the effect of material type on K values and a Tukey post-hoc multiple comparisons test was used for mean comparisons, with the letters above each bar indicating significant differences between materials. The K value and D-value, derived from the linear decline analysis, were plotted against each other for each material in podzol (B) and loam (D) with a Pearson correlation coefficient used to determine the correlation between the values (shown as R, with the significance level shown as P in each plot and an ellipse used to indicate 95% confidence).
